# Deltaproteobacteria and Spirochaetes-Like Bacteria Are Abundant Putative Mercury Methylators in Oxygen-Deficient Water and Marine Particles in the Baltic Sea

**DOI:** 10.3389/fmicb.2020.574080

**Published:** 2020-09-22

**Authors:** Eric Capo, Andrea G. Bravo, Anne L. Soerensen, Stefan Bertilsson, Jarone Pinhassi, Caiyan Feng, Anders F. Andersson, Moritz Buck, Erik Björn

**Affiliations:** ^1^Department of Chemistry, Umeå University, Umeå, Sweden; ^2^Department of Aquatic Sciences and Assessment, Swedish University of Agricultural Sciences, Uppsala, Sweden; ^3^Institut de Ciències del Mar (ICM-CSIC), Barcelona, Spain; ^4^Department of Environmental Research and Monitoring, Swedish Museum of Natural History, Stockholm, Sweden; ^5^Centre for Ecology and Evolution in Microbial Model Systems – EEMiS, Linnaeus University, Kalmar, Sweden; ^6^Department of Gene Technology, Science for Life Laboratory, KTH Royal Institute of Technology, Solna, Sweden

**Keywords:** mercury methylation, hgcAB, Baltic Sea, deltaproteobacteria, spirochaetes-like bacteria

## Abstract

Methylmercury (MeHg), a neurotoxic compound biomagnifying in aquatic food webs, can be a threat to human health via fish consumption. However, the composition and distribution of the microbial communities mediating the methylation of mercury (Hg) to MeHg in marine systems remain largely unknown. In order to fill this knowledge gap, we used the Baltic Sea Reference Metagenome (BARM) dataset to study the abundance and distribution of the genes involved in Hg methylation (the *hgcAB* gene cluster). We determined the relative abundance of the *hgcAB* genes and their taxonomic identity in 81 brackish metagenomes that cover spatial, seasonal and redox variability in the Baltic Sea water column. The *hgcAB* genes were predominantly detected in anoxic water, but some *hgcAB* genes were also detected in hypoxic and normoxic waters. Phylogenetic analysis identified putative Hg methylators within Deltaproteobacteria, in oxygen-deficient water layers, but also Spirochaetes-like and Kiritimatiellaeota-like bacteria. Higher relative quantities of *hgcAB* genes were found in metagenomes from marine particles compared to free-living communities in anoxic water, suggesting that such particles are hotspot habitats for Hg methylators in oxygen-depleted seawater. Altogether, our work unveils the diversity of the microorganisms with the potential to mediate MeHg production in the Baltic Sea and pinpoint the important ecological niches for these microorganisms within the marine water column.

## Introduction

Methylmercury (MeHg) is a neurotoxic compound that accumulates in aquatic food webs and may be a threat to human health related to fish consumption (Mason et al., 2012). Methylation of inorganic mercury (Hg) to MeHg is predominantly a biological process driven by anaerobic bacteria and archaea carrying the *hgcA* and *hgcB* genes (Parks et al., 2013) and takes place in various oxygen-deficient environments (e.g., sediment, water, soil). Hg methylation appears to be controlled by the activity of Hg-methylating microbes, the composition and activity of microbial communities (that indirectly modulate Hg methylation), and Hg bioavailability (Bravo and Cosio, 2019). It is broadly established that the capacity for Hg-methylation is limited to specific microbial lineages, with the most commonly reported groups found in the Deltaproteobacteria and Methanomicrobiales (Gilmour et al., 2013; Podar et al., 2015; Bravo et al., 2018; Yu et al., 2018). However, recent work has expanded this view and unraveled a higher phylogenetic diversity of microbes carrying the *hgcAB* genes than previously expected (McDaniel et al., 2020) calling for further analyses of microbial Hg-methylation in aquatic environments.

Recent advances in metagenomics have yielded new insights into the microbial taxonomic and functional diversity in various aquatic ecosystems (e.g., Mehrshad et al., 2016; Haro-Moreno et al., 2018; Nowinski et al., 2019). The approach has for example been applied to broadly assess the presence and diversity of genes central to biological Hg cycling in marine systems (Podar et al., 2015; Gionfriddo et al., 2016, 2020; Bowman et al., 2019; Lin et al., 2020; Tada et al., 2020; Villar et al., 2020). Podar et al. (2015) only detected *hgcAB* genes in a few metagenomes from marine pelagic waters (seven out of 138 metagenomes) but highlighted that limited sequencing depths of these metagenomes could have hampered detection. While a more recent study did not detect *hgcA* genes in waters from the Arctic and equatorial Pacific Oceans (Bowman et al., 2019), the presence of *hgcAB*-like genes was reported in normoxic water from the open ocean in several ocean basins (Villar et al., 2020), the mesopelagic zone of the East China Sea (Tada et al., 2020) and Antarctica sea ice (Gionfriddo et al., 2016), with a fraction of those genes being associated to a microaerophilic nitrite oxidizing bacteria. Further, Blum et al. (2013) demonstrated that between 20 and 40 % of MeHg measured in surface mixed layer of the North Pacific Ocean originated from internal production in the surface water. Finally, recent work from Gorokhova et al. (2020) reported the presence of *hgcA* genes from Deltaproteobacteria and Firmicutes in the gut microbiome from copepods collected in normoxic Baltic Sea water, suggesting that endogenous Hg methylation in zooplankton may be one path of Hg transfer from lower to higher trophic levels in this aquatic system.

Oceans and coastal areas, such as the West Coast of South America, the Arabian Sea, The Gulf of Mexico and the Baltic Sea, have experienced increased decline in oxygen since at least the middle of the 20th century (Breitburg et al., 2018). This phenomenon can be caused by (i) warming that decreases the solubility of oxygen in the ocean and (ii) nutrient enrichment in coastal water causing an increase of algal biomass and subsequent decomposition of sinking organic matter by microbes consuming the oxygen (Breitburg et al., 2018). Such oxygen deficient waters potentially offer ecological niches suitable for Hg-methylating microorganisms. Overall there are still important knowledge gaps concerning the process of Hg methylation in aquatic systems, in particular regarding the influence of variable redox conditions. In addition, marine particles (organic-rich particulate matter and aggregates) is hypothesized to provide both substrates for heterotrophic microbes (Azam and Long, 2001; Azam and Malfatti, 2007) and various anaerobic microenvironments (Alldredge and Silver, 1988; Bianchi et al., 2018) that could potentially favor Hg methylation, via e.g., microbial sulfate-reduction. Based on this, several studies proposed (Monperrus et al., 2007; Cossa et al., 2009; Sunderland et al., 2009; Lehnherr et al., 2011; Schartup et al., 2015) or demonstrated (Ortiz et al., 2015; Gascón Díez et al., 2016). Hg methylation in marine particles. For the Baltic Sea it has been proposed that Hg methylation in normoxic water can be associated with phytoplankton blooms via production of increased levels of phytoplankton derived OM that is also manifested as particulate organic particles sinking through the water column (Soerensen et al., 2016) that may providing suitable anoxic niches for Hg methylators. However, as far as we know there are no studies on microbial communities in relation to this phenomenon in the Baltic Sea or elsewhere.

The Baltic Sea is an ecosystem that has experienced large increases in nutrient loads and oxygen consumptions over the last century, resulting in extensive coastal and offshore zones with permanent hypoxic and anoxic water below the oxygenated surface water (Conley et al., 2011; Carstensen et al., 2014). As such, the Baltic Sea represents a model for the expansion of coastal ecosystems influenced by anoxia. Elevated MeHg concentrations in the Baltic Sea have been observed in anoxic water (>1000 fM) compared to hypoxic and normoxic water (Kuss et al., 2018; Soerensen et al., 2018). Soerensen et al. (2018) demonstrated that this was caused by increased rates of Hg methylation in the oxygen deficient water zones. They hypothesized that this process is predominantly driven by microbial sulfate-reduction because of the relatively high concentrations in dissolved sulfide in the anoxic water (up to ∼60 μM S^–II^). Although the concentrations of MeHg in normoxic water were generally low (13–80 fM), concentrations were higher than what could be explained by MeHg input from external sources only, and the authors could infer *in situ* formation of MeHg at a low rates also in normoxic water zones in the Baltic Sea (Soerensen et al., 2016, 2018). The presence and distribution of microorganisms carrying the *hgcAB* genes, including taxa known to reduce sulfate, could unequivocally confirm the potential for *in situ* MeHg formation in the Baltic Sea.

In this study, we assessed the spatial and seasonal variability of *hgcAB* genes in the Baltic Sea, including water column profiles, allowing us to investigate for the first time the presence and variation of Hg-methylating microbes across redoxclines in the Baltic Sea. We show the presence and relative abundance of Hg methylators in both hypoxic and anoxic water masses of the Baltic Sea, while *hgcAB genes* were present in very low abundance or not at all detected in normoxic waters. In addition, we found a higher proportion of *hgcAB* reads in metagenomes obtained from 3 μm filters compared to 0.2 μm filters, suggesting particles as an important habitat for Hg methylators. Our work provides new information on Hg-methylating microorganisms in coastal seas impacted by oxygen deficiency.

## Materials and Methods

**FIGURE 1:**
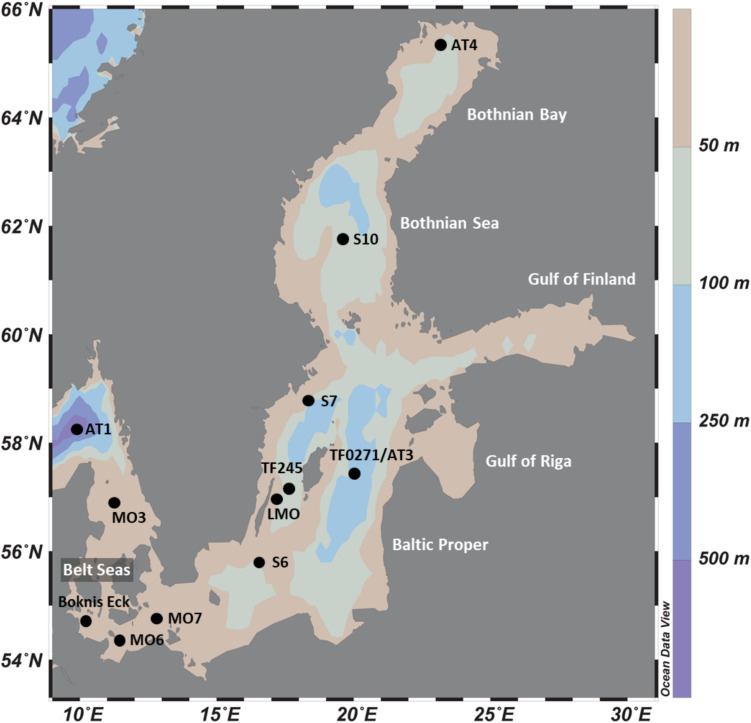
Location of study sites in the Baltic Sea.

The Baltic Sea Reference Metagenome (BARM) data (Alneberg et al., 2018) used in our study is composed of 81 metagenomes combined from three datasets spanning 13 locations (Figure 1) and are selected to cover natural variation in geography, depth and seasons of Baltic Sea waters. A summary on sampling and filtration of water samples for each of the datasets is provided in Table 1. We classified the water samples into three categories based on the measured O_2_ concentrations: (i) normoxic water with O_2_ concentrations exceeding or equal to 2 mL O_2_.L^–1^ (ii) hypoxic water with detectable O_2_ concentrations lower than 2 mL O_2_.L^–1^ and (iii) anoxic water with no detectable O_2_.

**TABLE 1:** Description of the number of metagenomes obtained with their respective sampling and filtration strategies for each of the three datasets.

Sample set name	Number of samples	Sampling	Filtration
*Redoxcline 2014*	14 samples	Samples were collected from normoxic, hypoxic and anoxic water at (i) Gotland deep (TF0271 station) on October 18 2014 (2 samples), October 26 2014 (8 samples) and (ii) Boknis Eck station on September 23 2014 (4 samples)	Six samples were filtered on 3 μm without pre-filtration and six samples were filtered on a 0.2 μm filter using 3 μm filter for pre-filtration (Oct 26 and Sep 23 samples). The two samples collected Oct 18 were captured on a 0.2 μm filter without pre-filtration.
*Transect 2014*	30 samples	Samples were collected from normoxic and hypoxic waters (from 2 to 242 m depth) from June 4 to June 17, 2014 at ten stations (AT1, AT3, AT4, MO3, MO6, MO7, S6, S7, S10, TF245).	The 30 samples were captured on a 0.2 μm filter without pre-filtration
*LMO 2012 time series*	37 samples	Surface waters (2 m depth) were sampled from the Linnaeus Microbial Observatory (LMO) station east of Öland between March 14 and December 20, 2012.	The 37 samples were captured on a 0.2 μm filter with pre-filtration using 3 μm filter

Methods for DNA extractions, library preparations and sequencing as well as the initial processing of metagenomics data is described in greater details in Alneberg et al. (2018). Briefly, preprocessed reads were co-assembled using Megahit (version 1.0.2, Li et al., 2015). Functional and taxonomic annotation was performed for the 6.8 million genes found on the 2.4 million contigs > 1 kilobase. To determine the overall composition of bacterial and archaeal communities, we used the metagenomics data analyzed in Alneberg et al. (2018) and compiled in BalticMicrobeDB^1^.

In order to detect *hgcAB* homologs genes, we first analyzed the 6.8 million predicted genes with the hmmsearch program from the hmmer (3.2.1 version, Finn et al., 2011) with the HMM profiles of concatenated *hgcAB* amino acid sequences from Podar et al. (2015) as a reference database (Supplementary Datasheet S1). We considered genes with *E*-values ≤ 10^–3^ as significant hits resulting in a total of 3,215 genes. Only a fraction of these 3,215 genes correspond to *hgcA* and *hgcB* genes and we therefore performed a manual check of their amino acid sequences using the knowledge from the seminal paper of Parks et al. (2013) that described unique motifs in *hgcA* (NVWCA(A/G/S)GK) and *hgcB* amino acid sequences (C(M/I)EC(G/S)(A/G)C). To ensure the reliability of the outputs obtained using the HMM profiles of *hgcAB* amino acid sequences from Podar et al. (2015) to capture all *hgcAB*-like genes from the metagenomic dataset, we applied the same procedure using the HMM profiles provided by the ORNL hgcAB library (Gionfriddo et al., 2020). The two procedures resulted in the recovery of the same *hgcA* genes and *hgcAB* gene clusters from the BARM dataset.

For phylogenetic analysis, we used the amino acid sequences of the 650 *hgcAB* gene clusters identified as putative Hg methylators by McDaniel et al. (2020). These 650 *hgcAB* gene clusters were obtained from the analysis of publicly available isolate genomes and metagenome-assembled genomes (Supplementary Datasheet S2, McDaniel et al., 2020). For each phylogenetic analysis, amino acid sequences were aligned using MUSCLE (cluster method UPGMA) in the software MEGAX (Kumar et al., 2018) and approximate maximum likelihood (ML) trees were constructed using FastTree (Price et al., 2009) with JTT+CAT model of sequence evolution and 1,000 ultrafast bootstrap replicates. The trees were visualized using iTOL (Letunic and Bork, 2019) and clades were collapsed by the dominant, monophyletic phyla when possible for visualization ease.

## Results

### Detection of hgcAB Genes Across the Baltic Sea Metagenomes

**FIGURE 2:**
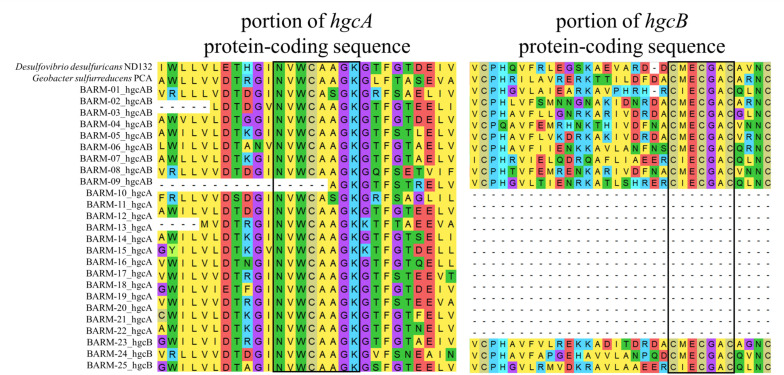
Alignment of conserved regions for amino acid sequences of the nine *hgcAB*-like gene clusters, (BARM-01 to 09) 13 *hgcA*-like genes (BARM-10 to 22), three *hgcB*-like genes (BARM-23 to 25) detected in the BARM dataset. The corresponding *hgcAB* amino acid sequences from *Desulfovibrio desulfuricans* ND132 and *Geobacter sulfurreducens* PCA were added in the alignment as references.

The total DNA sequence counts (number of reads) in the 81 Baltic Sea metagenomes ranged from 1.6 to and 37 million reads (mean 12.9, sd: 9.7). Among the total of 6.8 million protein-coding genes predicted from the co-assembly, 22 *hgcA*-like and 12 *hgcB*-like genes were detected. In some cases, *hgcA* and *hgcB* genes were found side-by-side on the same contig. Overall, we detected: (i) nine *hgcAB*-like gene clusters, (ii) thirteen *hgcA*-like genes, and (iii) three *hgcB*-like genes (Figure 2, Supplementary Datasheet S3). The resulting 25 gene clusters or single genes were named as displayed in Figure 2. The thirteen *hgcA*-like genes found without a coupled *hgcB* gene were always found at an extremity of the respective contig, possibly explaining why no *hgcB* genes were detected alongside. In contrast, the three *hgcB*-like genes found alone on their respective contig were consistently found in the central portions of the contigs, with no downstream or upstream amino acid motifs from *hgcA* genes. Most of the *hgcA* and *hgcB* genes had the most common amino acid motifs, NVWCAAGK and CMECGAC, respectively, as described by Parks et al. (2013). Two gene clusters contained both the “NVWCASGK” and “CIECGAC” motifs (BARM-01 & -09) while BARM-07 was the only gene cluster with the NVWCAAGK and CIECGAC combination (Figure 2).

### Taxonomic Identifications of *hgcAB*-Like Genes Found in the Baltic Sea

**FIGURE 3:**
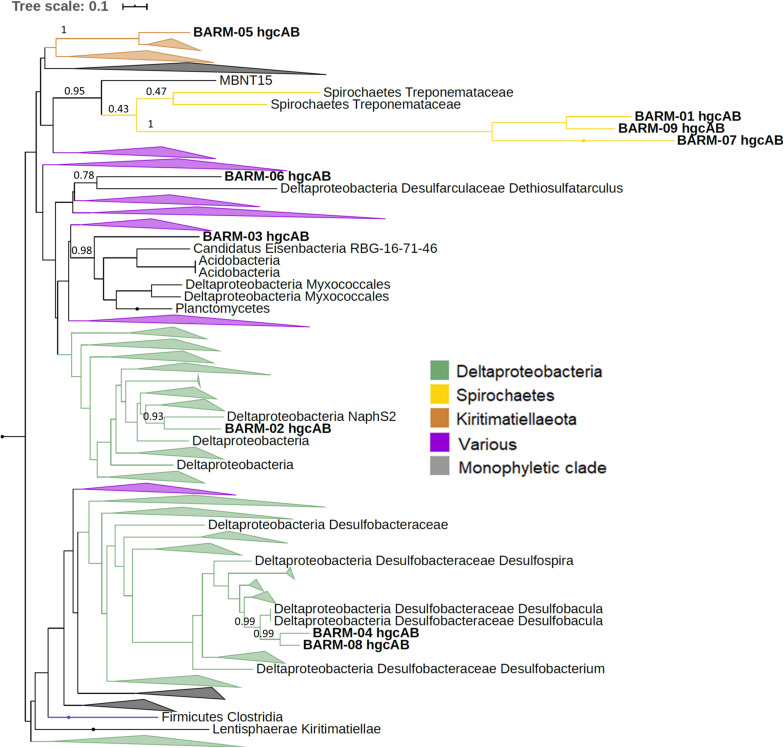
Maximum-likelihood phylogenetic tree of concatenated *hgcAB* amino acid sequences with collapsed clades. Collapsed clades are discriminated with colors based on their taxonomy. Gene clusters detected in the Baltic Sea water column (BARM) are displayed in bold. Bootstrap values obtained for nodes leading to BARM hgcAB amino acid sequences.

In order to taxonomically identify each Hg methylation gene detected in the BARM dataset, we constructed phylogenetic trees using the 650 *hgcAB* gene clusters generated by McDaniel et al. (2020). For the nine *hgcAB*-like gene clusters concatenated, we performed a phylogenetic analysis with the 650 *hgcAB* gene clusters (Figure 3, Supplementary Figure S1). For the 13 *hgcA* detected alone in their respective contig, an additional phylogenetic trees were performed using the 650 *hgcA* and 482 *hgcB* genes, respectively (Supplementary Figures S2, S3). The phylogenetic analysis revealed the presence of several *hgc* genes (referring hereafter either to *hgcAB* gene clusters, *hgcA* genes or *hgcB* genes) affiliated with the order Desulfobacterales (Deltaproteobacteria, class Desulfobacterota in GTDB classification): a member of the family Desulfobulbaceae (BARM-15 & -17), a *Desulfobacula* sp. (BARM-04 & -08), and a *Desulforhopalus* sp. (BARM-11) (Figure 3). In addition, the *hgc* genes BARM-02, -06 & -10 were associated with members of the orders Desulfatiglanales (naphS2 family) and Desulfarculales (Desulfarculaceae family) and Syntrophales. The *hgc* genes BARM-01, -07, -09 & -21 clustered together and were related – even if with very long branch length – to genes detected in various microbial phyla, with the closest related *hgc* genes detected in the genomes of two *Spirochaetes* from the family *Treponemataceae* and were referred thereafter as *hgcAB*-carrying Spirochaetes-like bacteria. Three *hgc* genes (BARM-05, -19 & -22) were associated with members of the recently discovered phylum Kiritimatiellaeota (Spring et al., 2016, defined as a class of Verrucomicrobia in GTDB classification), which is part of the widespread PVC superphylum (i.e., including Planctomycetes, Verrucomicrobia, Chlamydiae and Lentisphaerae). In addition, some BARM *hgc* genes were closely related to Firmicutes (Clostridia, BARM-14), a group of *hgc* genes from Euryarchaea and Chloroflexi clustered together (BARM-18, -24) and to member of the Spirochaetales order (BARM-25). Finally, seven BARM *hgc* genes were associated with clades including various microbial lineages and are thus classified here as unidentified (BARM-03, -12, -13, -16, -20, -23).

### Relative Abundance of *hgcAB* Genes in the Baltic Sea Water Column

**FIGURE 4:**
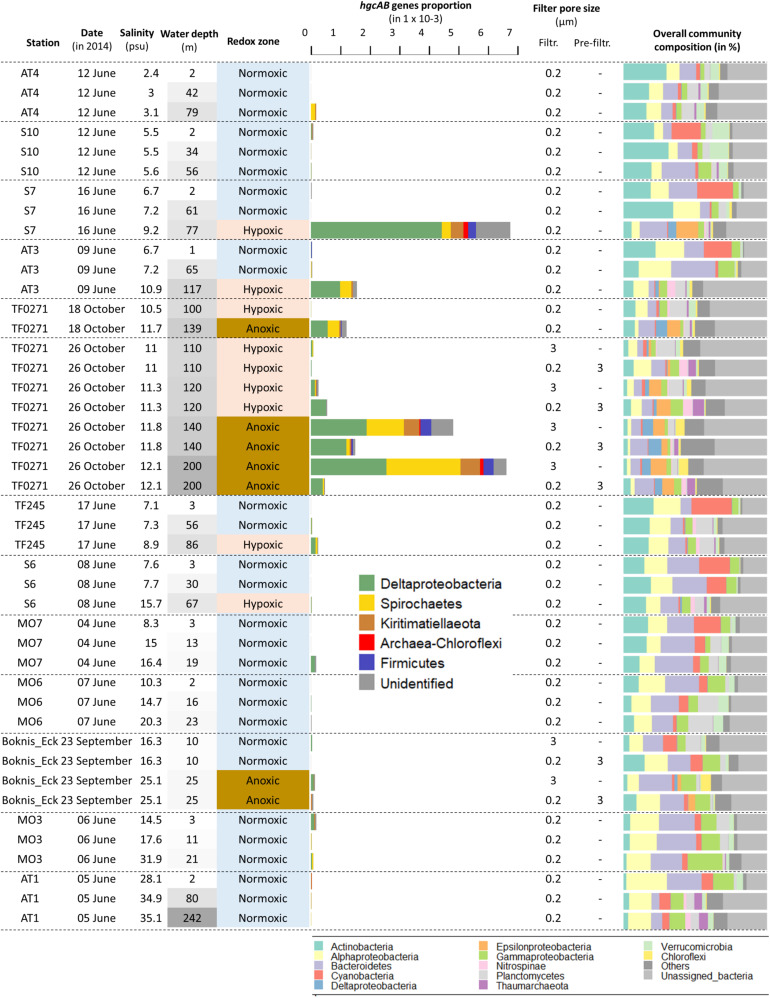
Relative abundance of *hgcA*- and *hgcB*-like genes in samples from the dataset “Redoxcline 2014” and “Transect 2014”. The dataset “LMO 2012” is not included because only few genes were detected and at low abundances (data shown in Supplementary Datasheet S4). The sampling date is displayed. Samples are ordered based on station locations from Bothnian Bay to Belt Seas (see Figure 1). The sampling depth (m) is color coded based on its respective gradient (darker shade of gray with increasing depth). The water redox zone is color coded based on the O_2_ categories defined in the text (light blue for normoxic, beige for hypoxic and brown for anoxic conditions). The abbreviations “Filtr.” and “Pre-filtr.” indicate the pore size of the filters used to obtain each metagenome and if pre-filtration were done prior to filtration, respectively. The composition of microbial communities is described from the dominant bacterial and archaeal lineages in proportion of DNA sequences related to the total number of DNA sequences identified as bacteria and archaea in the metagenomes.

The summed relative abundance of *hgcA* and *hgcB*-like genes in the Baltic Sea water column (i.e., the number of reads for the *hgcAB*-like genes per total annotated reads per sample, expressed as %) ranged from undetected to 6.7 × 10^–3^ % (mean: 0.3 × 10^–3^; sd: 1.2 × 10^–3^) (Table 2, Figure 4). The highest relative abundance of *hgcAB*-like genes was found in the hypoxic water from 76.5 m depth at station S7 with 6.7 × 10^–3^ % (Figure 4). Elevated abundance of *hgcAB*-like genes was also found in hypoxic and anoxic water from the TF0271/AT3 station with the highest values at a water depth of 200 m with 6.5 × 10^–3^ %. The proportion of *hgcAB* genes detected in the other 10 locations was relatively low with a maximum value of 0.2 × 10^–3^ % in the hypoxic layer (87.5 m) at station TF245 (Figure 4). At the LMO station, for which only normoxic water was sampled, the highest proportion of *hgcAB* genes found in the 37 samples was less than 0.01 × 10^–3^ % (sample LMO 120806, Supplementary Datasheet S4). We found that the most abundant *hgcAB* genes in Baltic Sea anoxic water belonged to members of Deltaprotebacteria, more specifically members of Desulfobulbaceae, Desulfarculaceae, *Desulfobacula* sp. and Syntrophales, and to microbes closely related to Spirochaetes from the Treponemataceae family (Figure 4, Supplementary Datasheet S5). In terms of overall community composition, the metagenomes obtained from anoxic and hypoxic layers were dominated by Deltaproteobacteria, Epsilonproteobacteria, Chloroflexi and Thaumarchaeota. In contrast, metagenomes from normoxic water samples exhibited higher proportion of DNA sequences identified as Actinobacteria, Alpha- and Gammaproteobacteria, Bacteroidetes and Cyanobacteria (Figure 4).

**TABLE 2:** Proportion (units in 1 × 10^–3^; normalized to total DNA sequences count of each metagenome) of *hgcA*- and *hgcB*-like genes in the 81 metagenomes.

Samples	Mean	Standard deviation	Min	Max
normoxic water (*n* = 65)	<0.1	<0.1	0.0	0.2
hypoxic water (*n* = 9)	1.0	2.2	<0.1	6.7
anoxic water (*n* = 7)	2.1	2.5	<0.1	6.5
all water (*n* = 81)	0.3	1.2	0.0	6.7

*The values obtained from the metagenomes collected in the three type of water defined in this study (normoxic, hypoxic and anoxic water) are displayed in this table.*

### Differences in *hgcAB* Genes Relative Abundance With Filter-Size

The quantity of *hgcAB*-like genes detected in metagenomes obtained from the Baltic Sea water column differed systematically between filter size fractions (Figure 4). Metagenomes obtained from the TF0271 station profile, and filtered onto 3 μm filters (hereafter referred to as the “3 μm metagenomes”), had consistently higher proportions of *hgcAB*-like genes (up to 6.5 × 10^–3^ %) than metagenomes obtained from 0.2 μm filters following pre-filtration with 3 μm filters (hereafter referred to as the “0.2–3 μm metagenomes”) (up to 1.5 × 10^–3^ %) (Figure 4). The relative abundance of *hgcAB* genes from the anoxic TF0271-Oct26 samples was three and 14 times higher in 3 μm metagenomes compared to 0.2–3 μm metagenomes for samples collected at 140 and 200 m depth, respectively (Figure 4, Supplementary Datasheet S4). At Boknis Eck, only a few samples were collected and the gene proportions were too low to properly investigate differences between filtration methods. At TF0271, *hgcAB* genes from Deltaproteobacteria were predominant for both 0.2–3 and 3 μm metagenomes (Figure 4). In these metagenomes, the most abundant *hgcAB* genes were attributed to Deltaproteobacteria from the families Desulfobulbaceae and Desulfarculales (Supplementary Datasheet S4). The 3 μm metagenomes from anoxic TF0271 samples (both 140 and 200 m) featured higher proportions of genes affiliated with Spirochaetes-like and Kiritimatiellaeota-like bacteria (Figure 4). In terms of overall community composition, the main differences between 3 μm metagenomes and 0.2–3 μm metagenomes was higher proportions of Chloroflexi in 3 μm metagenomes and of Bacteroidetes and Thaumarchaoeta in 0.2–3 μm metagenomes (Figure 4). Interestingly, the Kiritimatiellaeota proportion in hypoxic and anoxic water metagenomes from station TF0271/AT3 were more abundant in 3 μm metagenomes than in 0.2–3 μm metagenomes (Supplementary Datasheet S5). In contrast, Spirochaetes were found with same proportion in both types of metagenomes for hypoxic and anoxic water at TF0271 (Supplementary Datasheet S5).

## Discussion

Our phylogenetic analysis of the Baltic Sea Reference Metagenome dataset (Alneberg et al., 2018) identified at least 18 different *hgcAB* gene clusters that belong to several microbial lineages (Figure 3, Supplementary Figures S2, S3). The total number of DNA sequences obtained for each metagenome was relatively low (12.9 ± 9.7 million DNA sequences) compared to the numbers typically obtained in nowadays metagenomic studies (e.g., >20 million DNA sequences per metagenone) and therefore a higher sequencing depth may have provided a more refined picture of the diversity of *hgcAB*-carrying microbes in the Baltic Sea water as shown by the hgcAB-like genes rarefaction curves obtained from the BARM dataset (Supplementary Figure S4). The majority of *hgcAB* genes detected were affiliated with Deltaproteobacteria (or Desulfobacterota with GTDB classification) notably in genomes from possible sulfate reducing bacteria (e.g., Compeau and Bartha, 1984, 1985; Gilmour et al., 2013). Some of the identified Deltaproteobacteria/Desulfobacterota belong to groups of organisms previously known or predicted to perform Hg methylation, such as a species of *Desulfobacula* (Gionfriddo et al., 2016), members of the Desulfobulbaceae family (Benoit et al., 2001; Colin et al., 2018), and a member of the naphS2 group (Parks et al., 2013). It is noteworthy that none of the *hgcAB* genes detected in the water of the Baltic Sea were closely related to known iron reducing bacteria such as the Geobacteraceae family (Fleming et al., 2006; Kerin et al., 2006; Bravo et al., 2018). Relatively high Fe(II) concentrations have previously been reported in anoxic water from the Baltic Sea (>25 nM; Pohl and Fernández-Otero, 2012) although there is seasonal and inter-annual variability in concentrations. Our finding that none of the *hgcAB* was closely related to Geobacteraceae suggests that other iron reducing bacteria without the capability to methylate Hg, such as *Shewenella* sp. (incl *S. baltica*), are responsible for the formation of Fe(II) in the Baltic sea water column. Indeed, Shewanellaceae are in general one to two orders of magnitude more abundant than Geobacteraceae in the metagenomes compiled in the BalticMicrobeDB (Supplementary Datasheet S5; see text footnote 1). Our results thus suggest that Hg methylation is not linked to iron-reduction in the Baltic Sea. Recent studies also identified *hgcAB*-carrying Nitrospirea/Nitrospinae from several environments including marine systems (Gionfriddo et al., 2016; Tada et al., 2020; Villar et al., 2020). Despite members of this genus having been detected in open ocean and seas, we did not find any *hgcAB*-like genes related to *Nitrospina* sp. in any Baltic Sea water sample. In support of this, eleven metagenome-assembled genomes (MAGs) of Nitrospinae were extracted from the Baltic Sea metagenomes (Alneberg et al., 2020), and none of these contained *hgcAB*-like genes (data not shown).

Microbial syntrophy, defined as an obligate mutualistic metabolism, is a process known to occur mainly in environments with shortage of favorable electron acceptors, e.g., mostly in anoxic environments (McInerney et al., 2009; Morris et al., 2013). Some syntrophic microbes were recently suggested to be involved in the Hg methylation process (Gilmour et al., 2013; Yu et al., 2018; Hu et al., 2020), but we did not identify *hgcAB* genes from known syntrophic microorganisms in the Baltic Sea. Instead, we identified *hgcAB*-carrying bacteria closely related to various groups that are still poorly described (McDaniel et al., 2020) including members of Spirochaetes and Kiritimatiellaeota phyla. For instance, among the predominant *hgcAB*-like genes detected in Baltic Sea, the *hgcAB* genes BARM-01, -07 & -09 were clustered together and found close to a number of Spirochaetes (Figure 3). However, it should be noted these gene clusters, that formed very long branches in the inferred phylogenetic tree, are in a badly resolved region of the tree also containing MBNT15, Acidobacteria, Actinobacteria and Verrucomicrobia (Supplementary Figure S1 for full description), which precludes a safe identification at present. Although, Kiritimatiellaeota phylum is not well described (Spring et al., 2016), strains from this group were recently identified as anaerobic degraders of sulfated polysaccharides compounds (Van Vliet et al., 2019). Such compounds are produced by marine algae and are generally abundant in marine waters (Helbert, 2017). The availability of sulfated polysaccharides could explained the presence of Kiritimatiellaeota in oxygen-deficient water samples from TF0271 and S7 stations.

The 81 metagenomes collected from three sampling efforts (Alneberg et al., 2018) cover substantial variations in water depth, season and location across the Baltic Sea and our study investigates the relationship between putative Hg methylating populations and these factors. We found higher relative abundance of *hgcAB* genes in oxygen deficient water (hypoxic and anoxic layers) compared to those observed in normoxic layers also when including an extensive time series (37 time points over the year 2012) at 2 m depth. While no MeHg concentrations were measured alongside with the 81 samples used in the present study, Soerensen et al. (2018) reported MeHg concentrations measured in samples collected from several stations in the Baltic Sea in September 2014 and we compared such data with the proportion of the *hcgAB*-like genes detected in the metagenomes. For instance, they reported relatively high MeHg concentrations (787 fM) in an anoxic sample collected at 200 m depth at the station TF0271/AT3 (named BY15 in their work) for which high proportion of *hgcAB*-like genes was also found in our study (Soerensen et al., 2018; Figure 4). This finding is in agreement with the general understanding that methylation of Hg in aquatic systems is associated with anoxic conditions (Compeau and Bartha, 1984; Gilmour et al., 1992; Eckley et al., 2005; Malcolm et al., 2010; Bravo et al., 2015). We also found relatively high proportion of *hgcAB*-like genes in hypoxic water samples at two locations (TF0271/AT3, 117 m and S7 77 m depth) for which MeHg concentrations in Soerensen et al. (2018) were also high (i) 459 fM at 125 m depth at TF0271/AT3 station (named BY15 station in their work) and (ii) 1625 fM at 70 m and 1375 fM at 80 m depth at S7 station (named BY32 station in their work). By contrast, very low abundance of *hgcAB*-like genes were found at Boknis Eck station in the anoxic water layer (25 m). We hypothesized that the absence of putative mercury methylators in this anoxic water layer is caused by its strong seasonality in oxygen concentrations (Lennartz et al., 2014) inducing a strong turnover of microbial communities that does not provide suitable conditions for the establishment of stable mercury methylating communities. It is noticeable that several studies (Goñi-Urriza et al., 2015; Bravo et al., 2016; Christensen et al., 2019) have demonstrated a poor quantitative relation between Hg methylation rate and the presence of *hgcA* genes (mRNA and DNA), likely because of additional important factors/processes affecting Hg methylation. Therefore the very low abundance of *hgcAB*-like genes in Bocknis Eck anoxic layer do not necessarily indicate the absence of mercury methylation. MeHg concentrations measured in locations near our studied stations were always found to be relatively low consistent with low relative abundances of *hgcAB*-like genes. Overall, the comparison between the presence of MeHg and hgcAB-like gene suggests that both anoxic and hypoxic water layers from the Baltic Sea are conducive environments for Hg methylation.

*In situ* formation of MeHg in normoxic waters has previously been proposed to occur in anaerobic microzones such as around marine particles such as organic-rich particulate matter and aggregates (Monperrus et al., 2007; Cossa et al., 2009; Sunderland et al., 2009; Lehnherr et al., 2011; Schartup et al., 2015) and recently in zooplankton gut (Gorokhova et al., 2020). In some cases this has been experimentally demonstrated (Ortiz et al., 2015; Gascón Díez et al., 2016). Our study expands on the role of settling particles for Hg methylation in coastal seas and support the concept of settling particles as important habitats for Hg methylators also in anoxic waters. Firstly, we found higher relative abundances of *hgcAB* genes in the metagenome of marine particles from anoxic (3 μm filters) compared to hypoxic (3 μm filters) or normoxic (not pre-filter with 3 μm filters) water samples (Figure 4). Considering that the 3 μm filters represent the particle and aggregated organic matter fraction, our results suggest that marine particles becomes a more suitable habitat for Hg methylators (and thus potentially constitutes an environment with high Hg methylation rate) when they reach the anoxic zone. This phenomenon is likely caused by an increased prevalence of anaerobic conditions in the marine particles from anoxic water layers. Secondly, we found a higher proportion of DNA sequences of *hgcAB* genes in 3 μm metagenomes than in the free-living microbes, represented by the metagenomes obtained from 0.2 to 3 μm size fraction, in anoxic waters. This finding could be explained by marine particles featuring high concentrations of organic compounds that are suitable electron donors and fuel microbial activities occurring in anoxic conditions (Bianchi et al., 2018). It is noticeable that previous studies demonstrated differences between particle-associated and free-living bacterial communities in the Baltic Sea water column, but Spirochaetes, for which *hgcAB*-like genes closely related to previously described *hgcAB*-carrying Spirochaetes were found relatively abundant in the 3 μm metagenomes from BARM dataset, were not identified as being more predominant in particle-associated bacterial communities (Rieck et al., 2015, Supplementary Datasheet S5). In contrast, Kiritimatiellaeota were found more abundant in 3 μm metagenomes than 0.2–3 μm metagenomes in Baltic Sea data (Supplementary Datasheet S5), and this is consistent with their *hgcAB*-genes predominance in marine particles found in our study.

Our findings advance the understanding of the diversity and distribution of genes involved in Hg methylation as well the *hgcAB*-carrying microbial populations in marine environments. Particularly noteworthy was the finding that most of *hgcAB*-carrying microbes in the Baltic Sea water column were Deltaproteobacteria/Desulfobacterota predominantly found in oxygen-deficient zones (anoxic but also hypoxic zones). In addition, the differences in the relative abundance of *hgcAB* genes in metagenomes obtained from 0.2 compared to 3 μm pore size filters suggest that marine particles could be a preferred niche for Hg methylating microbial communities and therefore and important potential hotspot for MeHg formation in both hypoxic and anoxic sea water zones. Finally, our phylogenetic analysis highlights that a substantial part of the Hg methylators present in the Baltic Sea, and likely in other marine environments, are still poorly described and more works are needed to isolate, characterize and describe their genetic diversity.

## Data Availability Statement

Publicly available datasets were analyzed in this study. This data can be found here: https://doi.org/10.6084/m9.figshare.c.3831631.v1.

## Author Contributions

EC, MB, and EB designed this work. EC and MB performed the data analysis. All authors contributed to the data interpretation and writing of the manuscript.

## Conflict of Interest

The authors declare that the research was conducted in the absence of any commercial or financial relationships that could be construed as a potential conflict of interest.

## Footnotes

1 https://figshare.com/collections/BARM_Annotation_and_Quantification_Data/3831631

## Supplementary Material

FIGURE S1 Maximum-likelihood phylogenetic tree of concatenated *hgcAB* amino acid sequences of 650 *hgcAB* gene clusters (McDaniel et al., 2020) and BARM *hgcAB* genes. Gene clusters detected in the Baltic Sea water column (BARM) are displayed in bold. Bootstrap values are displayed aside each node (1,000 ultrafast bootstrap replicates).

FIGURE S2 Maximum-likelihood phylogenetic tree of concatenated *hgcA* amino acid sequences of 650 *hgcA* gene clusters (McDaniel et al., 2020) and BARM *hgcA* genes. Gene clusters detected in the Baltic Sea water column (BARM) are displayed in bold. Bootstrap values are displayed aside each node (1,000 ultrafast bootstrap replicates).

FIGURE S3 Maximum-likelihood phylogenetic tree of concatenated *hgcA* amino acid sequences of 482 *hgcB* gene clusters (McDaniel et al., 2020) and BARM *hgcA* genes. Gene clusters detected in the Baltic Sea water column (BARM) are displayed in bold. Bootstrap values are displayed aside each node (1,000 ultrafast bootstrap replicates).

FIGURE S4 Rarefaction curves of *hgcAB*-like gene sobtained from the BARM dataset.

DATASHEET S1 HMM profiles of *hgcAB* amino acid sequences modified from Podar et al. (2015).

DATASHEET S2 Amino acid sequences of *hgcAB* gene clusters identified in 650 bacterial and archaeal genomes (McDaniel et al., 2020).

DATASHEET S3 Amino acid sequences of *hgcAB*-like genes detected in BARM dataset.

DATASHEET S4 Proportion of *hgcAB* genes (out of the total number of reads of each metagenome) in each metagenome from the three datasets used in the present study. The taxonomic identifications associated to each gene are displayed. The environmental conditions associated to the water samples for each metagenome are displayed.

DATASHEET S5 Proportion of DNA sequences identified to bacterial and archaeal groups for each metagenome from the three datasets used in the present study. The taxonomic identifications associated to each gene are displayed. The environmental conditions associated to the water samples for each metagenome are displayed. Fine taxonomic resolutions is displayed for orders and families from Deltaproteobacteria.
