# Supplementary figures and images for: Deltaproteobacteria and Spirochaetes-Like Bacteria Are Abundant Putative Mercury Methylators in Oxygen-Deficient Water and Marine Particles in the Baltic Sea
Source: Front Microbiol. 2020 Sep 22;11:574080. doi: 10.3389/fmicb.2020.574080 (PMC7536318; doi:10.3389/fmicb.2020.574080)

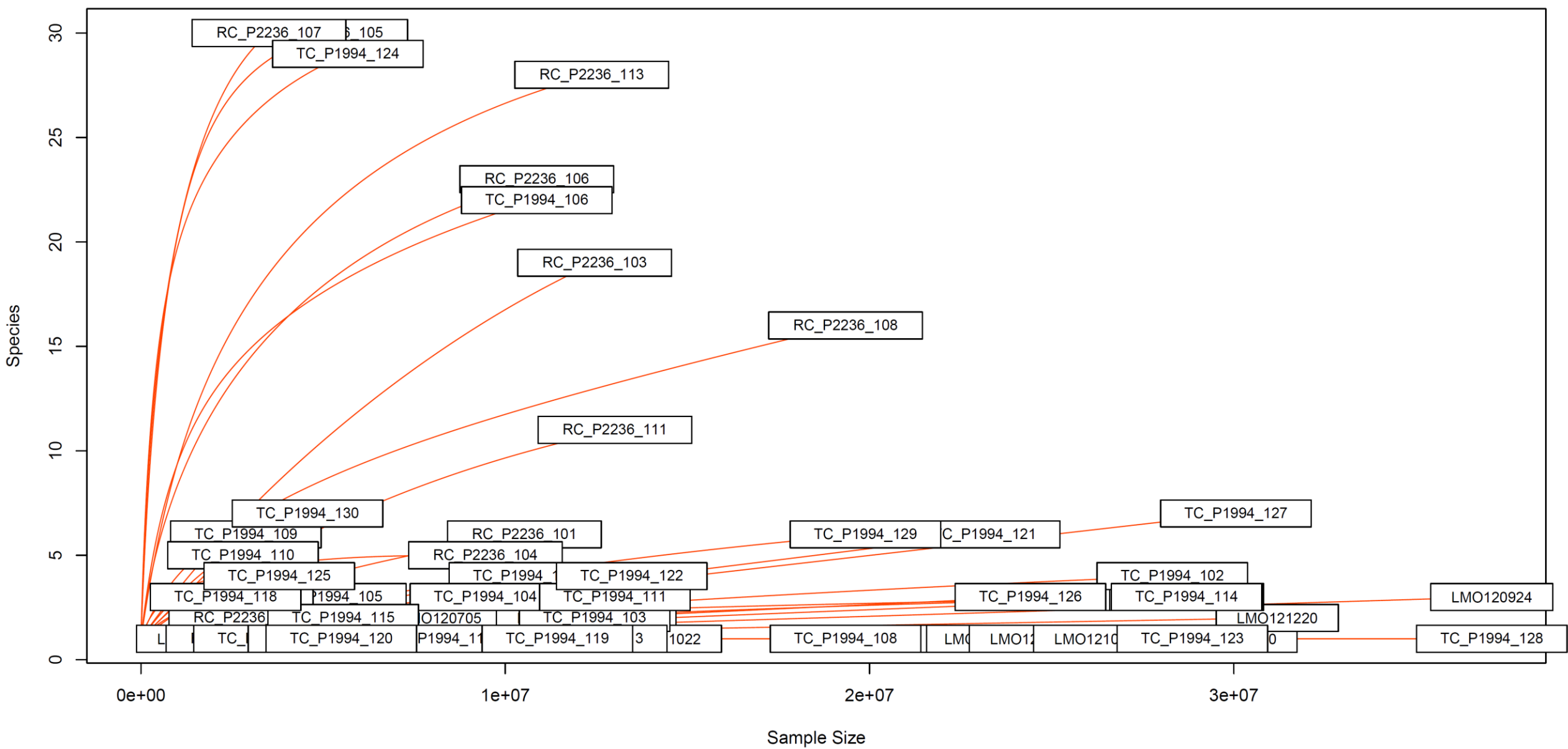

Supplement: FIGURE S4 — Rarefaction curves of hgcAB-like gene sobtained from the BARM dataset. [file Data_Sheet_4.PDF]
