# Supplementary material for: Deltaproteobacteria and Spirochaetes-Like Bacteria Are Abundant Putative Mercury Methylators in Oxygen-Deficient Water and Marine Particles in the Baltic Sea
Source: Front Microbiol. 2020 Sep 22;11:574080. doi: 10.3389/fmicb.2020.574080 (PMC7536318; doi:10.3389/fmicb.2020.574080)
Supplement: FIGURE S2 — Maximum-likelihood phylogenetic tree of concatenated hgcA amino acid sequences of 650 hgcA gene clusters (McDaniel et al., 2020) and BARM hgcA genes. Gene clusters detected in the Baltic Sea water column (BARM) are displayed in bold. Bootstrap values are displayed aside each node (1,000 ultrafast bootstrap replicates). [file Data_Sheet_2.PDF]

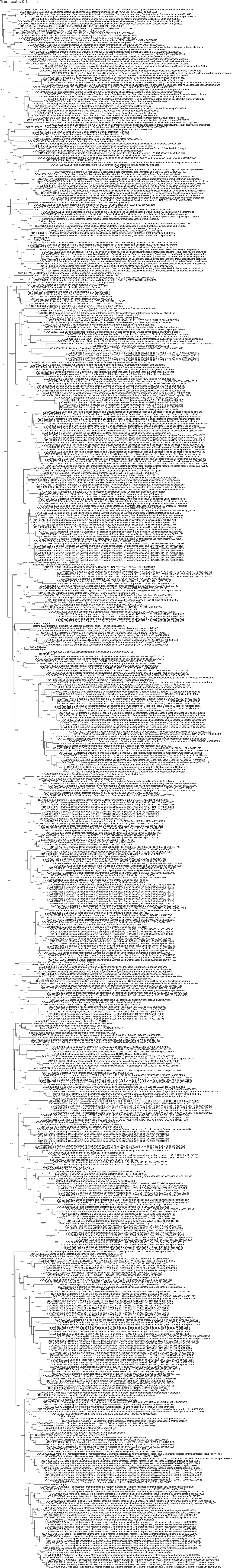
Tree scale: 0.1
